# A coronary artery anomaly: type IV dual left anterior descending artery

**DOI:** 10.5830/cvja-2010-032

**Published:** 2010-08

**Authors:** MURAT CELIK, ATILA IYISOY, TURGAY CELIK

**Affiliations:** Department of Cardiology, Gulhane Military Medical Academy, School of Medicine, Ankara, Turkey; Department of Cardiology, Gulhane Military Medical Academy, School of Medicine, Ankara, Turkey; Department of Cardiology, Gulhane Military Medical Academy, School of Medicine, Ankara, Turkey

**Keywords:** coronary artery anomaly, type IV dual left anterior descending artery

## Abstract

Coronary artery anomalies are seen in about 1.3% of patients undergoing coronary angiography. However, the dual type of left anterior descending (LAD) artery is a rare form of coronary artery anomaly. There are four types of dual LAD; type IV describes the anomaly of a rudimentary LAD artery terminating in the mid-portion of the anterior interventricular sulcus, and the presence of another LAD originating from the right coronary artery and continuing to the anterior interventricular sulcus.

## Introduction

Congenital anomalies such as origin, course and distribution of coronary arteries occur in 0.64 to 1.3% of patients undergoing coronary angiography. Although 80% of these coronary anomalies are benign, 20% may cause symptoms.^1^

The left anterior descending (LAD) artery courses along the anterior interventricular sulcus (AIVS) towards the cardiac apex, and anomalies of this coronary artery are extremely rare. The presence of a short and long LAD in the AIVS is described as a dual LAD. The short LAD travels and terminates in the AIVS and does not reach the cardiac apex, whereas the long LAD, which originates either from the left main coronary artery (LMCA) or the right coronary artery (RCA), enters the distal part of the AIVS and reaches the cardiac apex.^2^ There are four types of dual LAD. Type IV dual LAD differs from the first three types in the origination of the long LAD from the RCA. In this report, we present a case of type IV dual LAD.

## Case report

A 56-year-old woman with a two-year history of hyperlipidaemia was admitted to our hospital because of chest pain, especially during exercise. The physical examination and resting electrocardiogram (ECG) were normal and transthoracic echocardiography showed no wall-motion abnormality. Also, there was no abnormality in the standard biochemical tests. Her treadmill exercise test showed 1 to 2 mm down-sloping ST-segment depression in leads V4 to V6. Subsequently, the patient underwent coronary angiography for the evaluation of coronary artery disease.

Coronary angiography revealed a coronary artery anomaly of type IV dual LAD with a short LAD, which originated from the LMCA and terminated in the mid-portion of the AIVS. The long LAD originated from the RCA, entered into the distal part of the AIVS and travelled towards the apex of the heart. There was no haemodynamically severe stenosis in her coronary arteries and a decision was made to treat medically. The patient was discharged in good condition without any complications.

## Discussion

Coronary artery anomalies have become more relevant since the widespread application of coronary angiography. Interventional cardiologist should therefore be more familiar with these anomalies in order to make a more accurate diagnosis. Dual LAD is one of these coronary artery anomalies.

The presence of two coronary arteries in the AIVS is described as dual LAD. Spindola-Franco and colleagues3 reported that the incidence of dual LAD was about 1% in normal hearts and most of the patients were asymptomatic. Nevertheless, this anomaly can be found relatively often in patients with congenital heart disease such as tetralogy of Fallot and complete transposition of the great arteries.^4^ The anomalous origin of the left circumflex artery from the right coronary artery, associated with type IV dual LAD has also been reported.^5^

Spindola-Franco and co-workers3 reported an angiographic description of dual LAD and classified it into four subtypes according to the origin and course of the long LAD. Type I: the long LAD courses in the AIVS, descends on the left ventricular side of the short LAD, and then re-enters at the distal part of the AIVS. Type II: the long LAD courses in the AIVS, descends on the right ventricular side of the short LAD, and then re-enters at the distal part of the AIVS. Type III: the long LAD courses intramyocardially proximally in the ventricular septum, and appears on the epicardial surface in the distal part of the AIVS. Type IV: the long LAD unusually originates from the right coronary artery and then enters the AIVS.

In our patient, the long LAD originated from the RCA and entered the distal part of the AIVS. Therefore the diagnosis of type IV dual LAD, according to the classification of Spindola- Franco and colleagues,^3^ was made.

Type IV dual LAD is a rare congenital anomaly of the coronary arteries and it may cause misdiagnosis, and mistreatment when diagnosed. When a short or a hypoplastic LAD is present in the proximal part of the AIVS, type IV dual LAD should be kept in mind. The exact description of coronary artery anatomy is essential in patients undergoing percutaneous coronary intervention or coronary artery bypass graft (CABG) operations.^6^

The short LAD can be misdiagnosed as a total occlusion of the LAD; however, the diagnosis of dual LAD (type IV) can be made by the absence of retrograde flow and the detection of a second coronary artery originating from the RCA towards the AIVS. Also, the long LAD originating from the RCA can be misdiagnosed as a conus branch. But it should be kept in mind that it differs from a conus branch by the presence of septal and diagonal branches. Furthermore, there is a risk of incorrect placement of the graft in a CABG operation, so the recognition of a dual LAD may also increase the success rate of surgery.^4^

**Fig. 1.**
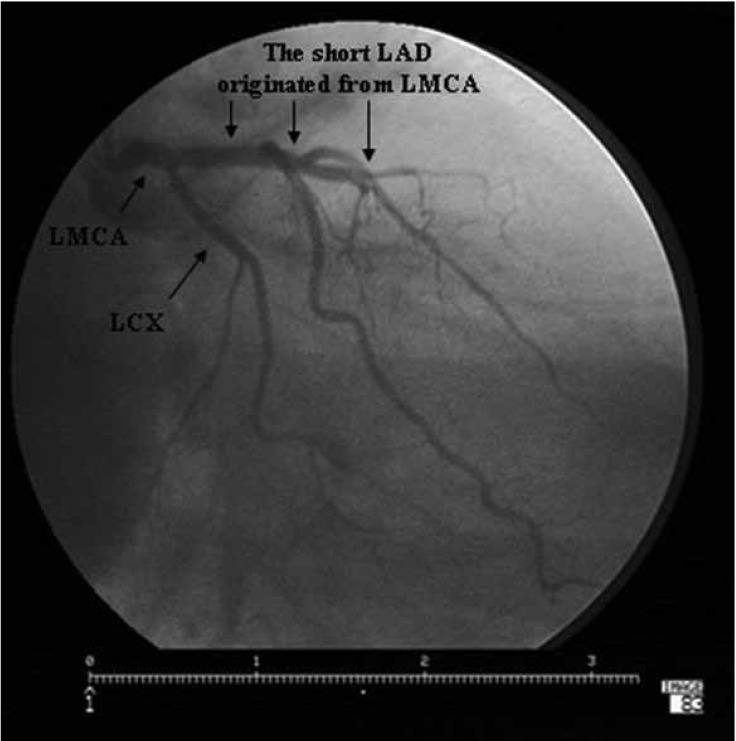
The short LAD originating from the LMCA terminated in the middle part of the AIVS . LMCA: left main coronary artery, LAD : left anterior descending artery, LCX: left circumflex coronary artery, AIVS : anterior interventricular sulcus.

**Fig. 2.**
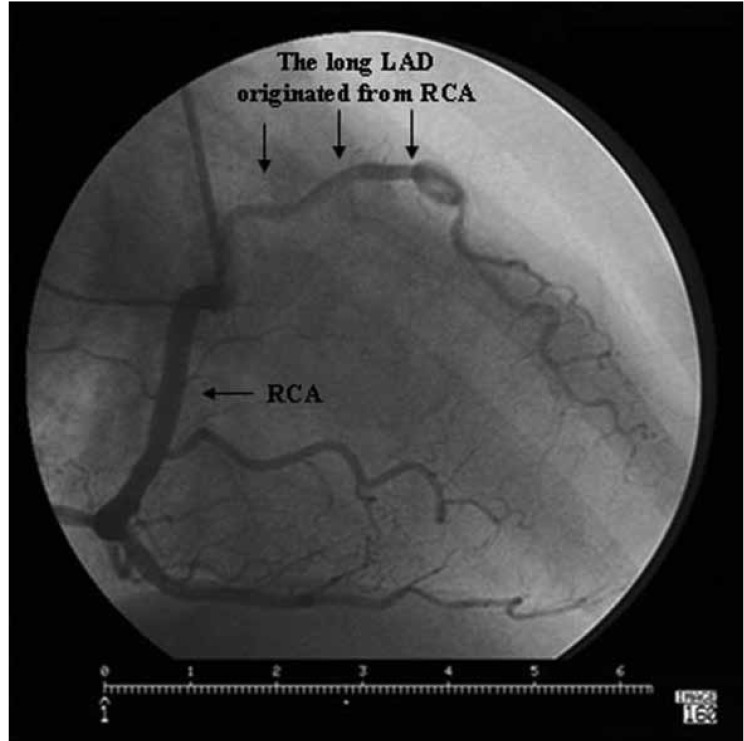
he long LAD originating from the RCA traversed the right ventricular infindubulum and entered the distal part of the AIVS . LAD : left anterior descending artery, RCA: right coronary artery, AIVS : anterior interventricular sulcus.

## Conclusion

The LAD is the most important coronary artery and being aware of the nature, anatomy and congenital anomalies of the LAD may help physicians make the correct diagnosis and treatment in patients undergoing percutaneous coronary intervention or CABG operations.
